# RNA-Seq and lipidomics reveal different adipogenic processes between bovine perirenal and intramuscular adipocytes

**DOI:** 10.1080/21623945.2022.2106051

**Published:** 2022-08-08

**Authors:** Xiaoyu Wang, Chengcheng Liang, Anning Li, Gong Cheng, Feng Long, Rajwali Khan, Jianfang Wang, Yu Zhang, Sen Wu, Yujuan Wang, Ju Qiu, Chugang Mei, Wucai Yang, Linsen Zan

**Affiliations:** a College of Animal Science and Technology, Northwest A&F University, Yangling, Shaanxi, China; b National Beef Cattle Improvement Center, Northwest A&F University, Yangling, Shaanxi, China; c Department of Livestock Management, the University of Agriculture, Peshawar, Pakistan; d Longri Breeding Farm of Sichuan Province, Sichuan, Chengdu, China; e Qinghai Academy of Animal Science and Veterinary Medicine, Qinghai University, Qinghai, Xining, China

**Keywords:** Beef, marbling, adipogenic, transcriptome, lipid dynamics

## Abstract

Adipogenesis involves complex interactions between transcription and metabolic signalling. Exploration of the developmental characteristics of intramuscular adipocyte will provide targets for enhancing beef cattle marbling without increasing obesity. Few reports have compared bovine perirenal and intramuscular adipocyte transcriptomes using the combined analysis of transcriptomes and lipid metabolism to explore differences in adipogenic characteristics. We identified perirenal preadipocytes (PRA) and intramuscular preadipocytes (IMA) in Qinchuan cattle. We found that IMA were highly prolific in the early stages of adipogenesis, while PRA shows a stronger adipogenic ability in the terminal differentiation. Bovine perirenal and intramuscular adipocytes were detected through the combined analysis of the transcriptome and metabolome. More triglyceride was found to be upregulated in perirenal adipocytes; however, more types and amounts of unsaturated fatty acids were detected in intramuscular adipocytes, including eicosapentaenoic acid (20:5 n-3; EPA) and docosahexaenoic acid (22:6 n-3; DHA). Furthermore, differentially expressed genes in perirenal and intramuscular adipocytes were positively correlated with the eicosanoid, phosphatidylcholine (PC), phosphatidyl ethanolamine (PE), and sphingomyelin contents. Associated differential metabolic pathways included the glycerolipid and glycerophospholipid metabolisms. Our research findings provide a basis for the screening of key metabolic pathways or genes and metabolites involved in intramuscular fat production in cattle.

## 1. Introduction

Intramuscular fat (IMF) deposition is closely related to the formation of marbling patterns [1] and is also important for the palatability of beef. Studies have shown that compared with subcutaneous and visceral adipocytes, the development of intramuscular adipocytes is different [2]. Therefore, the characteristics of intramuscular adipocyte development enable researchers to specifically enhance marbling without increasing overall animal obesity. Elucidation of IMF deposition mechanisms renders the design of nutritional or genetic methods possible for the modulation of processes involved and for changing the fatty acid composition of beef [3].

Adipogenesis is a process involving the establishment of complex interactions between transcriptional and metabolic signalling pathways [4]. A considerable portion of the knowledge on adipogenesis is derived from *in vitro* studies conducted using adipose-derived stem cells or preadipocytes. Such cell differentiation systems can be used in fundamental research to understand the coordinated relationship between cell proliferation and differentiation. Concurrently, they can also help reveal the regulatory mechanism involved in adipocyte development and may provide theoretical basis [5] for livestock breeding and related metabolic diseases. Researchers in the field have described the existence of two stages in adipogenesis. The first stage is known as determination. Cells are endowed with differentiation potential, and this phase depends on the specific phase of the cell cycle (contact inhibition), involving the orientation of pluripotent stem cells with the adipocyte lineage. The second stage is known as terminal differentiation. Preadipocytes that have acquired differentiation potential undergo differentiation into mature adipocytes under the stimulation of hormones and other inducing factors [4]. The commonly used adipocyte induction model MDI comprises 3-Isobutyl-1-methylxanthine (IBMX), dexamethasone (DEX), and insulin [6]. Under conditions of MDI stimulation, preadipocytes first enter a specific cell division phase (mitotic clonal expansion, MCE) and then undergo terminal differentiation to become adipocytes [7].

Metabolome research has been conducted to explore the differences in lipid composition between mice white adipose tissue (eWAT), beige adipose tissue (iWAT), and brown adipose tissue (iBAT) [8], as well as to investigate heat production associated with brown white adipose tissue [9,10]. Based on recently reported research methods, multi-omics joint analysis seems to be more effective in the study of the phenotypes and regulatory mechanisms of biological processes in biological models. The integrated analysis of transcriptome and lipidomic data also provides a new strategy in the study of fat deposition and lipid metabolism.

Thus far, few studies have compared the transcriptomes of bovine perirenal adipocytes and intramuscular adipocytes in the MCE phase. Additionally, much remains unknown regarding the lipidomics pathway which is involved in the differentiation of bovine adipocytes. This study focused on the early response of preadipocytes to adipogenic induction and late adipogenesis to explore the adipogenic differentiation of bovine perirenal and intramuscular preadipocytes. We performed a combined analysis of the transcriptome and metabolome in adipocytes, aiming to explore the regulatory network of PRA and IMA during both the MCE period and the late stages of adipogenesis. We also aimed to explore the differences in metabolic levels between PRA and IMA after differentiation. This was conducted to screen for adipocyte differentiation markers. Our study provides a theoretical basis for targeting the pathways involved in the regulation of beef cattle fat deposits in different parts of the body.

## 2. Materials and methods

### 2.1 Isolation and culture of bovine preadipocytes

In this experiment, all cell-based *in vitro* studies were repeated at least three times. Three 4-day calves were selected from the National Beef Cattle Improvement Centre Experimental Farm (Yangling, Shaanxi, China). For bovine intramuscular preadipocyte isolation, the longissimus dorsi muscle of the back at the 12/13 ribs was separated under anti-septic conditions, and then taken to the cell culture laboratory [11]. Samples were washed using PBS (Hyclone, UT, USA) with twice the amount of penicillin and streptomycin (Hyclone) three times. Tissues were cut into 1 ~ 2 mm^3^ tissue blocks, then digested with collagenase II (Sigma, MO, USA) for 90 min in 37°C water shaker. In this process, the concentration of collagenase II was 2 mg/mL, 5 ml of collagenase II was used to digest 5 g of tissue per depot. The digestive solution was neutralized with medium containing 10% FBS (GIBCO, NY, USA), and then filtered with 70 and 200 mesh stainless steel screens. The collected filtrate was centrifuged at 1500 rpm for 10 min, and the supernatant was discarded. The cells were resuspended in DMEM/F12 medium containing 10% FBS (growth medium). Cells were seeded in a petri dish (Corning, NY, USA) at a density of 2.5 × 10^5^ cells. Dishes were placed in a 5% CO_2_ incubator for 1.5 h, then washed with sterile PBS to remove non-adherent cells, and the medium was replaced with fresh growth medium. Cells were routinely cultured in a 37°C, 5% CO_2_ incubator.

For bovine perirenal preadipocyte isolation, samples were taken from bovine perirenal fat. The tissue was minced and digested with collagenase I(Sigma, MO, USA), than the obtained cells were washed and seeded in cell culture dishes without differential adhesion [12]. Other conditions are the same as above.

### 2.2 Differentiation induction of bovine preadipocytes

When the cells reach contact inhibition, growth medium was replaced with induction medium consisting of DMEM/F12 with 10% FBS, 0.5 mmol/ L IBMX(Sigma), 1 μmol/ L DEX(Sigma), 5 μg/ml insulin, and 2 μmol/ L rosiglitazone (Sigma). After 2 days of induction treatment, cell culture medium was changed to maintenance medium consisting of DMEM/F12 with 10% FBS and 5 μg/ml insulin. Preadipocytes were induced to differentiate for a total of 12 days, and the maintenance medium kept cells until the end of differentiation. Media were changed every 2 d until 12 d.

### 2.3 Oil red O staining

The culture medium was removed and the cells were fixed with 4% paraformaldehyde (Solarbio, Beijing, China) for 30 min. After adding oil red O (Sigma) which was soluble in isopropanol for 10 min and rinsing with PBS, the oil red O staining results were observed and photographed under the microscope (Olympus, IX71, Tokyo, Japan). Dyeing areas were counted with the Image J software (National Institutes of Health, Bethesda, MD), and 3 images were used for analysis each time [13].

### 2.4 Cell immunofluorescence

Cells were fixed for 30 min by 4% paraformaldehyde at room temperature, and then washed with PBS. Cells were permeabilized with 0.1% BSA (Sigma) and 0.5% Triton X-100 (Sigma) for 60 min at room temperature. Cells were blocked by 3% BSA blocking solution for 60 min at room temperature. Then, incubated with primary antibody PPARG(diluted 1:200, ab45036, Abcam, Cambridge, U.K.), ZNF423 (diluted 1:100, LSBio, LS-B14339-50, WA, USA), Adiponectin (AdipoQ)(diluted 1:100, Bioss, bs-0471 R, MA, USA) overnight at 4°C. After the primary antibody was discarded, the cells were incubated with fluorescent secondary antibody at 37°C for 1 h and washing with PBST (Phosphate Buffered Saline with Tween-20) for 3 times. For nuclear dyeing, 0.001 mg/mL DAPI (Thermo Scientific, MA, USA) was added and kept cells in a dark place for 5 min.

Fluorescence was observed under a fluorescence microscope (Invitrogen, EVOS Auto2, WA, USA). Dyeing areas were counted with the Image J software, and 3 images were used for analysis each time.

### 2.5 EdU (5-ethynyl-2'-deoxyuridine) assay

Cells were seeded in a 24-well plate at a density of 4 × 10^3^ ~ 1 × 10^5^ per well, 24 hours before EdU assay [14]. In this experiment, the EdU cell proliferation detection kit (RiboBio Co., Ltd, Guangzhou, China) was used, and the experimental procedure followed the instructions provided by the manufacturer. Dyeing areas were counted with the Image J software, and 3 images were used for analysis each time.

### 2.6 RNA-seq analysis

**Figure 1.**
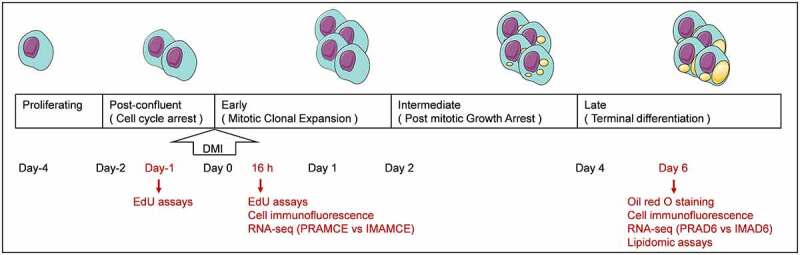
A scheme of chronological preadipocyte differentiations and sampling time points. After the induction of adipogenic differentiation by DMI, the contact-suppressed preadipocytes underwent several stages of differentiation, namely early, intermediate, and late stages. The day of induction was defined as Day 0. (5-ethynyl-2’-deoxyuridine) EdU assays were performed one day before induction(Day-1). EdU assays, cell immunofluorescence, and RNA-seq were performed 16 h after induction, and the groups were named PRAMCE and IMAMCE. Oil red o staining, cell immunofluorescence, RNA-seq, and lipidomic assays were performed on the 6th day after induction, and the groups were named PRAD6 and IMAD6.

RNA extraction and RNA-seq analysis were performed by NovoGene (Beijing, China), the sequencing platform was illumina novaseq 6000, and the read length was 150bp. The sampling time points were listed in Figure 1. FeatureCounts (1.5.0-p3) is used to calculate the reads mapped to each gene. Use DESeq2 software (1.16.1) to perform differential expression analysis between two comparative combinations (two biological replicates for each group). DESeq2 provides statistical procedures for determining differential expression in digital gene expression data using models based on the negative binomial distribution. Use the method of Benjamini and Hochberg to adjust the p value to control the false discovery rate. The reads count and RNA quality information of each sample were listed in Table S1. The selection criteria for differentially expressed genes (DEGs) are padj < 0.05, and |log2foldchange| > 0.5. The clusterProfiler (3.4.4) software was used to analyse the statistical enrichment of differentially expressed genes in the KEGG pathway.

### 2.7 Lipid sample preparation and lipidomic assay

Lipid extraction of perirenal and intramuscular adipocytes on the 6th day of differentiation and mass spectrometry-based lipid detection [15,16] were performed by Metware Biotechnology Co., Ltd. (Wuhan, China). The sampling time points were listed in Figure 1. Based on the UPLC-MS/MS detection platform (UPLC, Shim-pack UFLC SHIMADZU CBM A system, https://www.shimadzu.com/; MS, QTRAP® System, https://sciex. com/), use the the self-built database MWDB (Metware Biotechnology Co), and use the MRM detection mode to detect qualitatively and quantitatively of the lipid metabolites in biological samples. The multivariate statistical analysis process provided by Metware were used to analyse the difference between qualitative and quantitative lipid data. We analysed the metabolome data using a partial least squares-discriminant analysis (PLS-DA) model. Then, a variable importance in projection (VIP) value for an OPLS-DA model was obtained. We used a VIP > 0.5 and a |log2fold-change| ≥ 0.5 to screen for differential metabolites. Using the cor program in R, we calculated the Pearson correlation coefficients of each gene and metabolite and used nine quadrants to show that the Pearson correlation coefficient of each group was greater than 0.8. The different multiples of the metabolites of each gene were divided into 1–9 quadrants from left to right and from top to bottom using black dashed lines, as shown in Figure S1. Among them, quadrants three and seven indicated that the differential expression patterns of the genes and metabolites were consistent, and their regulation was positively correlated. This study was divided into two groups, PRAD6 and IMAD6 for metabolic research, with three biological replicates in each group.

### 2.8 Total RNA extraction and quantitative real-time PCR

Total RNA was extracted with an animal cell total RNA extraction kit (Tiangen, Beijing, China). RNA reverse transcription PCR was conducted using reverse transcription kits (Tiangen). TB Green Premix Ex Taq II kit (TaKaRa, Dalian, China) with CFX connect (Biorad, CA, USA) were used to complete the qPCR reactions. The data was analysed using the CFX Manager software package (Biorad), and the 2-ΔΔCt method was used for the calculations.

### 2.9 Statistical analysis

In this study, the statistical data are presented as mean ± SE (n = 3). The ANOVA program in SPSS Statistics (version 19.0; IBM) was used for analysis of variance. A comparison between the results of the two groups was conducted using the Student’s two-tailed t- test. A cut-off value of *p* < 0.05 indicated a significant difference, while *p* < 0.01 indicated a highly significant difference.

## 3. Results

### 3.1 Identification of preadipocytes

**Figure 2.**
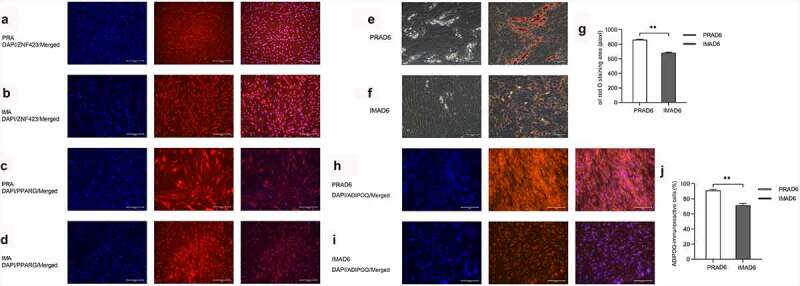
Preadipocyte identification (magnification: 100×). DAPI (blue) staining was performed to investigate the nucleus, and the right panel shows the merged view. **a**. ZNF423 (red) immunofluorescence in perirenal preadipocytes (PRAs). **b**. ZNF423 (red) immunofluorescence in intramuscular preadipocytes (IMAs). C. PPARG (red) immunofluorescence in PRAs. **d**. PPARG (red) immunofluorescence in IMAs. E. Presence of perirenal adipocyte lipid droplets and the results of oil red O staining. F. Presence of intramuscular adipocyte lipid droplets and the results of oil red O staining. **g**. Statistical results for an oil red O-stained area. **h**. Adiponectin (ADPN) immunofluorescence of PRAD6. **i**. ADPN immunofluorescence of IMAD6. DAPI (blue) staining was performed to investigate the nucleus, and the right panel shows the merged view. J. Percentage statistical results for ADPN-immunoreactive cells. Double asterisks (**) indicate highly significant variations (*p* < 0.01). Error bars represent SD (n = 3).

To detect the purity of preadipocytes isolated from bovine perirenal or intramuscular adipose tissue, the adipocyte surface antigen markers ZNF423 and PPARG [17] were used for conducting immunofluorescence assays. Results revealed that the proportion of ZNF423-immunoreactive cells among the PRA reached 69.8% ± 1.1%, whereas their proportion among the IMA reached 78.6% ± 0.8% Figure 2(a,b). The proportion of PPARG-immunoreactive cells among the PRA reached 74.8% ± 0.5%, and among the IMA, the proportion reached 71.3% ± 2.4% Figure 2(c,d). These results indicated that high-purity samples of preadipocytes were isolated, which were suitable for use in subsequent analyses.

To demonstrate the adipogenic ability of the isolated cells, the preadipocytes were induced to undergo adipogenic differentiation and were stained with oil red O. The lipid droplets of the PRA were observed to be larger and more abundant than those of the IMA Figure 2(e,f,g). Immunofluorescence was then conducted to detect the expression of Adiponectin (AdipoQ) in cells after differentiation. Visual analysis results showed that the proportion of AdipoQ immunoreactive cells among the differentiated PRA reached 91% ± 1.5%, whereas in the differentiated IMA, the proportion reached 71.3% ± 2.4%. In summary, it can be concluded that bovine perirenal preadipocytes possess a higher adipogenic ability than intramuscular adipocytes Figure 2(h,i,j).

### 3.2 During mitotic clonal expansion, bovine IMA showed higher proliferation abilities than PRA through the MAPK/PI3K/mTOR signalling pathway

**Figure 3.**
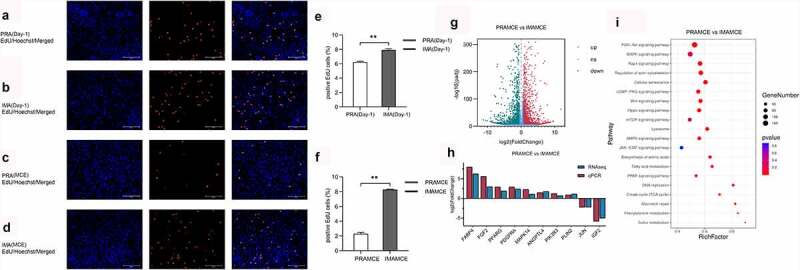
Analysis of the differentially expressed genes (DEGs) between perirenal preadipocytes (PRAMCE) and intramuscular preadipocytes (IMAMCE) during MCE. A, B. EdU assays conducted using perirenal preadipocytes/intramuscular preadipocytes (PRAs/IMAs) before confluency was achieved (Day-1, magnification: 100×). Cells during DNA replication were stained with EdU (red), and the nuclei were stained with the Hoechst stain (blue). C, D. EdU assays conducted using PRAs/IMAs after differentiation (MCE, magnification: 100×). E. Percentage statistical results for positive EdU cells before confluency was achieved (Day-1). F. Percentage statistical results for positive EdU cells after differentiation (MCE). Double asterisks (**) indicate highly significant variations (*p* < 0.01). Error bars represent SD (n = 3). G. DEGs observed between PRAMCE and IMAMCE during MCE. H. qPCR validation of the selected DEGs. I. KEGG enrichment of DEGs in the PRAs and IMAs during MCE.

**Figure 4.**
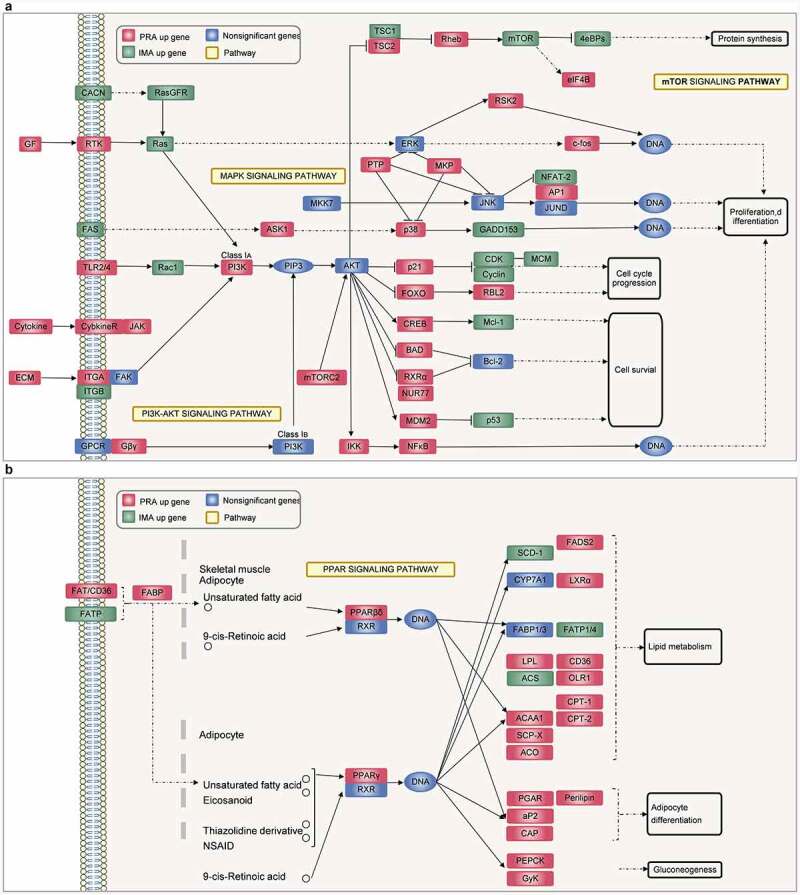
Adipocyte proliferation and adipogenesis in perirenal preadipocytes (PRAs) and intramuscular preadipocytes (IMAs) are regulated by different pathways during the specific cell division phase (MCE). A. Adipocyte proliferation in PRAMCE and IMAMCE is regulated by different pathways during MCE. B. Adipocyte adipogenesis in PRAMCE and IMAMCE is regulated through different pathways.

To compare the cell proliferation abilities of the PRA and IMA, we assayed the cells for EdU incorporation before cell confluency was achieved and 16 h after the induction of differentiation. Before reaching confluency, the proportion of PRA that were EdU-positive cells reached a proportion of 6.23% ± 0.10%, whereas for the IMA, the proportion reached 7.91% ± 0.16% Figure 3(a,b,e). We further compared the MCE period between the PRA and IMA in early adipogenic differentiation. Both PRA and IMA showed significant growth arrest before the induction of adipogenesis. Sixteen hours after the induction of adipogenesis, the proportion of IMA EdU-positive cells increased significantly (*p* < 0.01) compared to that of PRA EdU-positive cells. Figure 3 illustrates that the proportion of PRA EdU-positive cells reached 2.3% ± 0.20%, while that of IMA EdU-positive cells reached 8.27% ± 0.06% Figure 4(c,d,f). Results revealed that during MCE, IMA possessed higher proliferation abilities than PRA.

Simultaneously, the cells during this period were collected for RNA-seq. During the MCE process, it was found that bovine perirenal adipocytes (PRAMCE) and intramuscular adipocytes (IMAMCE) were regulated by different pathways. There were 2207 upregulated genes in the PRA group and 2103 upregulated genes in the IMA group Figure 3g. Gene ontology (GO) function enrichment analysis of the differentially expressed genes (DEGs) is mainly performed to examine the terms related to adipocyte proliferation and differentiation. Results showed that genes highly expressed in PRAMCE were enriched in relation to the regulation of the developmental process, the response to lipids, and the regulation of lipid storage (Figure S2A). However, genes that are highly expressed in the IMA are enriched in relation to system processes, cell population proliferation, and cell development (Figure S2B). We found that, during the MCE period, bovine perirenal adipocytes showed more positive responses to the induction of adipogenesis. Further enrichment analysis of the Kyoto Encyclopaedia of Genes and Genomes (KEGG) pathway showed that the main pathways enriched in the DEGs included PI3K-Akt, MAPK, Rap1, the regulation of the actin cytoskeleton, and various cellular senescence signalling pathways (Figure 3i, Table S2). Among these pathways, FABP4, FGF2, PPARG, as well as other genes were screened for quantitative PCR (qPCR) verification Figure 3h.

An integrated analysis of the DEGs and proliferation-related pathways revealed that the IMA showed a higher proliferation ability than the PRA through the MAPK/PI3K/mTOR signalling pathway Figure 4a. Notably, the expression of *GADD*153, *CDK*s, *Cyclins*, and other genes that are positively regulated in proliferation [18–20] were upregulated in the IMA, whereas the expression of negatively regulated factors such as p21 [21] were downregulated in the IMA. Concurrently, in terms of adipogenesis, the PPAR signalling pathway also showed a high degree of correlation with the PRA DEGs Figure 4b. Among the members of this axis, *PPARG, ACCA1, LPL, CD*36, and other genes related to adipogenesis were found to be highly expressed in the PRA. This shows that the PRA possess a higher fat-forming potential during the MCE period. The results showed that the transcriptional levels of adipogenic differentiation between bovine perirenal preadipocytes and intramuscular preadipocytes was markedly different and included changes in the expression of genes related to proliferation and early induction of adipogenesis.

### 3.3 Genes in lipid metabolic pathways are differentially expressed between PRA and IMA during adipogenic differentiation

**Figure 5.**
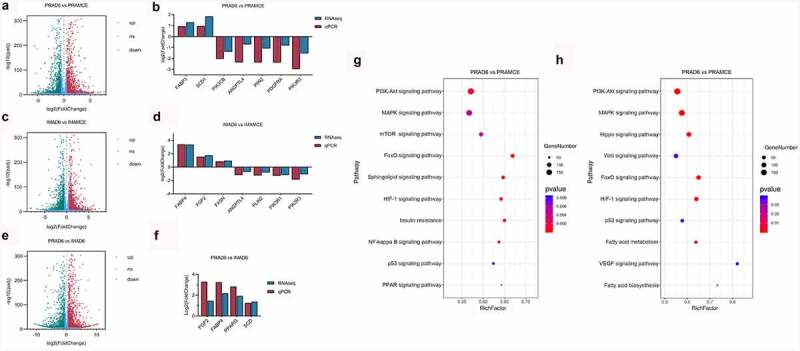
Adipocyte differentiation of PRAs and IMAs are regulated by different pathways. A. Differentially expressed genes (DEGs) during PRA differentiation. C. DEGs during IMA differentiation. E. DEGs between PRAD6 and IMAD6. B, D. F. qPCR validation of the selected DEGs. G. The KEGG enrichment pathway of the two groups of differential genes during PRA differentiation. H. The KEGG enrichment pathway of the two groups of differential genes during IMA differentiation.

RNA-seq was performed to explore the similarities and differences in transcription levels during adipogenic differentiation between bovine perirenal preadipocytes and intramuscular preadipocytes. This study focused on the early response to adipogenic induction and the adipogenic ability of PRA and IMA. Comparative time points were selected in the early stage of adipogenesis (MCE) and the late stages of adipogenesis (D6). A total of 19,146 genes were detected between PRAD6 and PRAMCE, including 2072 genes whose expression levels were upregulated after differentiation and 2082 genes whose expression levels were downregulated Figure 5a. A total of 19,153 genes were detected between IMAD6 and IMAMCE, including 1919 genes whose expression levels were upregulated after differentiation and 2101 genes whose expression levels were downregulated Figure 5c.

KEGG enrichment pathway analysis results obtained during differentiation showed that the PRA were mainly regulated by the PI3K-Akt, MAPK, mTOR, FoxO, and sphingolipid signalling pathways Figure 5g. However, the IMA were mainly regulated by the PI3K-Akt, MAPK, Hippo, Wnt, and FoxO signalling pathways Figure 5h. Results revealed that during adipogenic differentiation of the PRA and IMA, the enriched signalling pathways were different. Among these pathways, DEGs were related to adipogenesis were verified by qPCR Figure 5(b,d,f).

**Figure 6.**
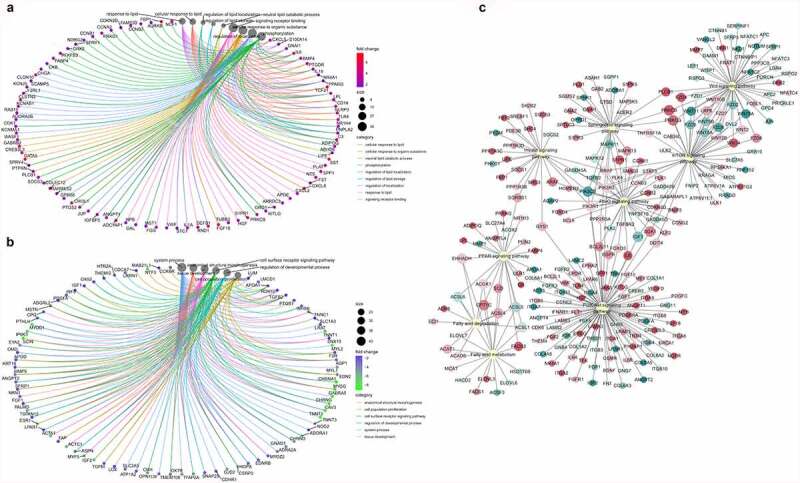
Functional analysis of the differentially expressed genes in bovine perirenal adipocytes (PRAD6) and intramuscular adipocytes (IMAD6) on the 6th day after differentiation. A. GO function enrichment analysis of DEGs upregulated in PRAD6. B. GO function enrichment analysis of DEGs upregulated in IMAD6. C. Enrichment of DEGs between PRAD6 and IMAD6 in pathways related to adipogenic differentiation and metabolism. The yellow circle indicates the enriched pathway. The red circles indicate genes upregulated in PRAD6, and the green circles indicate genes upregulated in IMAD6. The darker the colour, the greater the absolute value of log2FC. The larger the circle of a single gene, the more pathways that related to it.

A total of 19,375 genes were detected in the IMA and PRA after differentiation (PRAD6 and IMAD6). There were 2332 genes that were significantly upregulated in PRAD6, and 2015 genes that were significantly upregulated in IMAD6 Figure 5e. GO function enrichment analysis of the DEGs of PRAD6 and IMAD6 showed that the pathways related to lipid metabolism and accumulation were significantly enriched. Genes upregulated in PRAD6 were enriched in cellular response to lipids, regulation of lipid localization and regulation of lipid storage Figure 6a. Meanwhile genes upregulated in IMAD6 were enriched during system processes i.e. anatomical structure morphogenesis and tissue development Figure 6b. The KEGG pathway enrichment analysis revealed that the DEGs were significantly enriched in pathways related to adipogenic differentiation and lipid metabolism (Table S3). Among these, the pathways associated with adipogenic differentiation included the PI3K-Akt, PPAR, and FoxO signalling pathways. The pathways associated with metabolism included the sphingolipid signalling pathway, fatty acid metabolism, the mTOR signalling pathway, and fatty acid degradation Figure 6c. These results indicate that genes related to adipogenic differentiation and fatty acid metabolism are differentially expressed between PRA and IMA.

### 3.4 Comparative analysis of the lipid metabolism characteristics of bovine perirenal and intramuscular adipocytes

**Figure 7.**
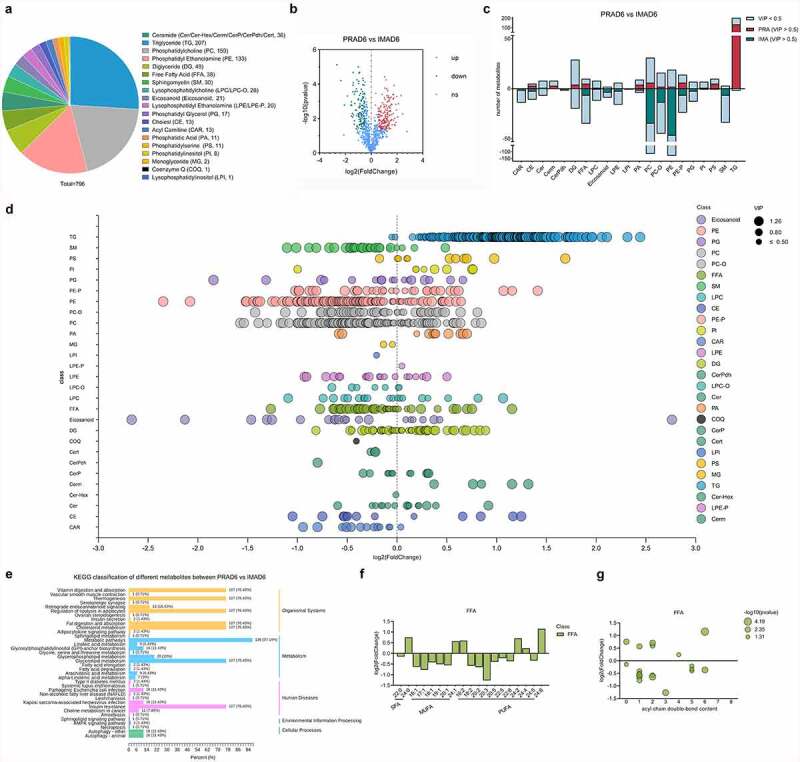
Lipid composition and changes detected in PRAD6 and IMAD6. A. Lipid contributions. B. Volcano map for the differential metabolites. C. Descriptive statistics for the different metabolites. D. Changes in metabolites between the two groups. E. Annotation to the KEGG database for significantly different metabolites (*p* ≤ 0.05). F. Composition analysis of the acyl chains of the FFAs. The abscissa indicates the number of carbon atoms in the acyl chain. The ordinate indicates the fold change of perirenal preadipocytes (PRAs) relative to intramuscular preadipocytes (IMAs). A log2fold-change > 0 indicates a metabolite with upregulated expression in the PRAD6 group, whereas a log2fold-change < 0 indicates that the metabolite expression was upregulated in the IMAD6 group. G. Double bond analysis of FFAs. The abscissa indicates the number of unsaturated bonds in the acyl chain. The ordinate indicates the fold change of PRAD6 relative to IMAD6.

Lipid metabolism is involved with the performance of essential functions necessary for growth and development, such as energy transport, communication between cells, and network regulation [22]. To expand the existing understanding of IMF deposition mechanisms, this study examined the lipid metabolism characteristics of bovine perirenal adipocytes and intramuscular adipocytes, and combined the findings with transcriptome sequencing analysis to explore the metabolic correlation network involving PRAD6 and IMAD6. Based on RRLC-MS/MS-based lipid metabolite detection, a total of 796 metabolites were detected. The types and amounts of metabolites detected are listed in Figure 7a. There were 179 upregulated metabolites in the PRA group and 135 upregulated metabolites in the IMA group Figure 7b.In the PRA group, the significantly upregulated metabolites included one hundred thirty-six types of triglycerides (TG), six types of PCs, four types of diglycerides (DG), and five types of free fatty acids (FFA). In the IMA group, the significantly upregulated metabolites included forty-seven types of phosphatidylethanolamines (PE), thirty-five types of PCs, seven types of sphingomyelins (SM), and seven types of FFA Figure 7c. The regularity of all detected lipid class changes is shown in Figure 7d. Particularly, there are more types and quantities of triglycerides in perirenal adipocytes, and there are more types and quantities of FFAs and glycerophospholipids in intramuscular adipocytes. Different metabolites were classified according to KEGG pathway annotation results.We found that the main enriched pathways were metabolic pathways, including glycerolipid metabolism and glycerophospholipid metabolism Figure 7e. This indicates that there are significant differences in the lipid composition of bovine perirenal adipocytes and intramuscular adipocytes.

The difference in fatty acid composition in beef can cause changes in its flavour [23]. This study further analysed and compared the composition of individual fatty acyl chains related to free fatty acids (FFAs) in perirenal adipocytes and intramuscular adipocytes. We selected FFA or fatty acyl chains that were significantly different (*p* ≤ 0.05), in terms of content, between PRAD6 and IMAD6 for analysis. The contents of 27 FFAs were significantly different between PRAD6 and IMAD6. As shown in figure 7f, one monounsaturated fatty acid (MUFA; C24:1) showed upregulated in PRAD6, and five MUFAs (C18:1, C19:1, C20:1, C16:1, and C17:1) showed upregulated in IMAD6. Upon comparing the polyunsaturated fatty acids (PUFA), it was found that there were four PUFAs (C16: 2, C24: 2, C24: 4, and C24: 6) whose contents were upregulated in PRAD6, and seven PUFAs (C22:5, C24:5, C22:6, C20:5, C18:2, C20:2, and C20:3) whose contents were upregulated in IMAD6. It was also found that DHA and EPA levels were significantly upregulated in IMAD6. Further analysis of the double bond contents in the FFAs Figure 7g showed that there were more types and higher quantities of unsaturated fatty acids in the IMA, particularly MUFA. These findings demonstrate that there are more types and higher quantities of unsaturated fatty acids in IMAD6 than in PRAD6. This was attributable to the differential expression levels of fatty acid storage and lipid metabolism-related genes.

Correlation analysis was performed for all the genes and metabolites detected in each group.To explore the characteristics of genes whose expression could positively regulate metabolites, the genes and metabolites have the same differential expression patterns were selected. The screening cut-offs were as follows: |log2fold-change| > 1 and padj < 0.05 for the differentially expressed genes, and VIP > 1 and |log2fold-change| > 1 for the related metabolites. Based on this finding, 2276 genes were identified. Results showed that the metabolites associated with these genes were mainly concentrated in the eicosanoid, PC, PE, and sphingomyelin. KEGG analysis was performed for the genes related to these four metabolites to explore the metabolic pathways in which eicosanoid (Figure S3A), PC (Figure S3C), PE (Figure S3B), and sphingomyelin (Figure S3D) were involved. GO enrichment analysis was performed for these genes. Among them, 68 genes were related to lipid metabolism, and the heat map reflecting their differential expression in IMAD6 and PRAD6 has been illustrated in Figure S3E. This indicates that the differentially expressed genes in IMAD6 and PRAD6 are positively correlated with the contents of the eicosanoid, PC, PE, and sphingomyelin.

### 3.5 Glycerolipid and glycerophospholipid compositions are different between PRAD6 and IMAD6

**Figure 8.**
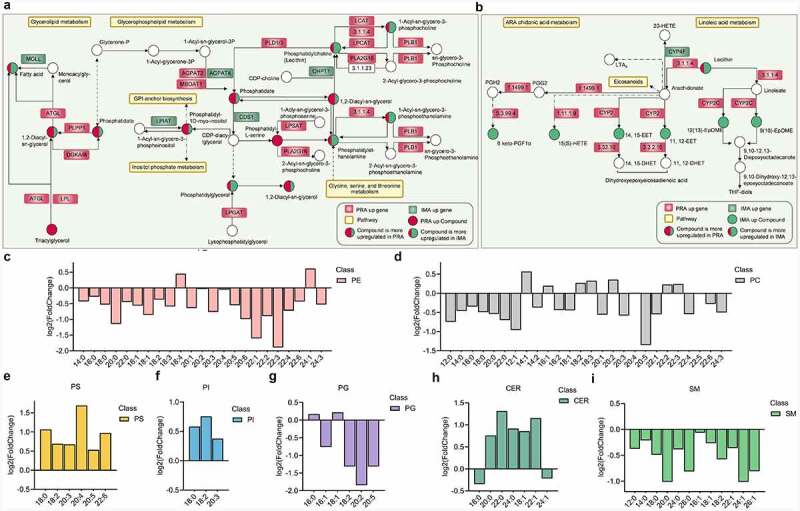
Comparison of glycerolipid, glycerophospholipid, and sphingomyelin compositions between bovine perirenal adipocytes and intramuscular adipocytes. A-B. Differential genes and metabolites in the pathway. C-E Composition analysis of the acyl chains of PE, PC, PS, PI, PG, CER, and SM. The abscissa represents the number of carbon atoms in a single fatty acyl chain related to different glycerophospholipids or sphingolipids. The ordinate represents the fold change of perirenal preadipocytes (PRAD6) relative to the intramuscular preadipocytes (IMAD6)

Based on the differential metabolites identified and the enrichment analysis results obtained for the differentially expressed genes (Table S3), Figures 8(a,b) were generated to illustrate the pathways that encompassed both the differential metabolites and the differential genes. The results of this study indicate that there are significant differences in the contents of glycerolipids and glyceryl phosphatide between bovine perirenal adipocytes and intramuscular adipocytes. There are higher quantities of TG, DG, phosphatidylserine (PS), phosphatidylinositol (PI), and phosphatidate (PA) in perirenal adipocytes. However, there are higher quantities of FFA, phosphatidyl ethanolamine (PE), PC, and phosphatidyl glycerol (PG) in intramuscular adipocytes. Figure 8a provides an overview of the glycerolipid and glycerophospholipid metabolisms and related genes selected from the KEGG analysis. Genes that play a key role in the synthesis of glycerol and glycerophospholipids exhibited significant differential expressions. This may be partly explain the significant differences observed in glycerolipid and glycerophospholipid contents between IMAD6 and PRAD6.

Furthermore, this study was conducted to analyse and compare the compositions of glycerophospholipids between the PRA and IMA, and a total of 183 different phospholipids were screened(*p* ≤ 0.05). Among them, 42 were upregulated in the PRA and 141 were upregulated in the IMA. An analysis of glycerophospholipids related to eicosapentaenoic acid (20:5 n-3; EPA) and docosahexaenoic acid (22:6 n-3; DHA) found that the increased phospholipid contents in the PRA included one containing EPA (20:5) and three containing DHA (22:6). Among the increased phospholipid contents in the IMA, eight contained EPA (20:5) and ten contained DHA (22:6). An analysis of the fatty acyl chain content of the glycerophospholipids found that in the PRA, C20:5 and C22:6 in PS increased significantly, whereas in the IMA, C20:5 in PE, PC, and PG increased significantly and C22:6 in PE and PC increased significantly. In general, more upregulated glycerophospholipid species were detected in the IMA, and higher EPA and DHA quantities were observed.

The metabolic pathways involved mainly include the glycerolipid, glycerophospholipid, ARA chidonic acid, and linoleic acid metabolisms. Although few metabolite classifications were conducted to detect significantly increased levels in both the PRAD6 and IMAD6 groups, these are not necessarily the same metabolites. In the present study, metabolites that were increased in greater numbers in PRAD6 or IMAD6 were distinguished (Table S4). In the glyceride metabolism pathway, expressions of all types of TGs detected in this study were upregulated in PRAD6, and correspondingly, higher ATGL and LPL gene expression levels were detected in PRAD6.

Sphingolipids are abundant membrane components and act as important signalling molecules in eukaryotic cells, which help to maintain the structure and function of the membrane [24]. Studies have shown that changes in sphingolipids are related to the development of metabolic diseases [25]. Expression levels of four types of ceramides (CER) were elevated in the PRA and two types were detected in the IMA(*p* ≤ 0.05) Figure 8(h). Our analysis conducted on fatty acyl chains showed that levels of C18:1, C20:0, C22:0, C22:1, and C24:0 increased in the PRA, whereas levels of 18:0 and 24:1 increased in the IMA. Sphingomyelin levels were elevated in the IMA, and our analysis conducted on fatty acyl chains also helped obtain the same result Figure 8(i). In sphingolipid metabolism, SM generates CER under the action of sphingomyelin synthase. Expression of sphingomyelin phosphodiesterase (*SMPD*1) was upregulated in the PRA, indicating that the increased expression of sphingomyelin phosphodiesterase in the PRA resulted in a lower SM content and a higher CER content(FigureH, I). Concurrently, in the PRA, expression levels of *SPTLC*3, *ACER*2, *ASAH*1, and other sphingolipid synthesis-related genes were significantly upregulated, while expression of *SPHK*1, which is related to sphingolipid breakdown, was significantly downregulated in the IMA. It is speculated that there exist more species and accumulation of sphingolipids increases in the PRA.

## 4. Discussion

In the present study, we performed RNA-seq and mass spectrometry-based lipidomics to expand our understanding of fat deposits arising from perirenal or intramuscular regions. Our results showed that PRA possessed higher adipogenic abilities than IMA. In the process of differentiation into mature adipocytes, adipocyte differentiation into the IMA and PRA is regulated through different pathways. More TG was detected to be upregulated in the PRA, but more types and amounts of unsaturated fatty acids, including DHA and EPA, were detected in the IMA.

### 4.1 Bovine perirenal and intramuscular preadipocytes exhibit different responses to induced adipogenesis in the early stages

MCE is an important stage after the induction of adipocyte differentiation [7]; however, studies have also shown that it is not a necessary step in the differentiation process of preadipocytes [26]. Insulin promotes cell growth and proliferation [27] and is the main component in the MDI that stimulates the clonal proliferation of pre-adipocytes in the contact inhibition period [26]. In the present study, IMA in the MCE period were highly prolific, and more proliferation-related genes were upregulated; however, IMA did not exhibit high differentiation abilities. We speculate that this may be attributable to the presence of IMA in the determination stage before the MCE, as more cells with adipogenic differentiation potential were not obtained at this stage, and the undifferentiated cells showed evident signs of being the proliferative phenotypes following insulin stimulation. We speculate that intramuscular adipocytes possess higher proliferation abilities than perirenal adipocytes, but perirenal adipocytes are more sensitive to adipogenic inducers, and thus differentiate into mature adipocytes rapidly. Previous studies have shown that the formation of visceral fat cells occurs between the middle of the foetal period and the early postpartum period [28]. It has also been reported that, based on systematic studies, intramuscular fat formation mainly occurs from the late stage of the foetal period to a gestational age of nearly 250 d in cattle. Therefore, there is a unique period during which marbling can be specifically enhanced without a concurrent increase in overall fat levels [3]. The adipocytes in this study were derived from 4-day-old calves. It is likely that perirenal adipocytes must have formed at this stage but intramuscular adipocytes did not form or less proportions might have formed. IMA may be derived from earlier progenitor cells, and PRA may be derived from specialized later cells. Furthermore, in the PPAR signalling pathway (related to adipogenesis during the MCE period), the upregulation of expression of adipogenic marker genes such as PPARG and FABP4 was detected and verified by qPCR. This corresponds to and is consistent with the later differentiation phenotype.

### 4.2 Differences in the metabolism of glycerolipids and phospholipids provide prospective targets for differential lipid deposition

This study shows that, based on the differential expression of fatty acid storage and lipid metabolism-related genes, there are more types and higher contents of unsaturated fatty acids in IMAD6 than those in PRAD6. This was found to be accompanied by the differential expression of fatty acid storage and lipid metabolism-related genes. Additionally, the genes that play a key role in the synthesis of glycerol and glycerophospholipid in IMAD6 and PRAD6 are expressed differently, which may be part of the reason for the significant differences observed in the contents of glycerol and glycerophospholipid in IMAD6 and PRAD6. Regulation of gene expression or controlling dietary composition to regulate the expression of candidate genes and the uptake and utilization of glycerides and glycerophospholipids by intramuscular fat cells will provide theoretical guidance for regulating the content of intramuscular fat and its fatty acid composition.

The transcription processes involved in the glyceride and glycerophospholipid metabolisms differ between bovine perirenal adipocytes and intramuscular adipocytes. This is accompanied by differences in the composition of glycerides and glycerophospholipids. Phospholipid phosphatase 1 (*PLPP*1) regulates cell signalling by adjusting the concentration of lipid phosphates relative to their dephosphorylated products [29]. In the present study, expression of the PLPP1 gene was found to be upregulated in PRAD6, and the types of PA and DG that were found to be upregulated in PRAD6 were present in higher quantities than those in IMAD6. Owing to the increase in the expression of monoglyceride lipase (*MGLL*) in IMAD6, more upregulated FFA species were detected in IMAD6. Phosphatidate (PA), as a lipid second messenger, is a key component of the plasma membrane where it is involved in cell signal transduction and membrane remodelling [30]. In the enriched pathways, we found that Phosphatidate(PA) was mainly derived from 1,2-diacyl-sn-glycerol (DG), 1-acyl-sn-glycerol-3P (LPA), and phosphatidylcholine (PC). The key enzymes involved in these pathways are diacylglycerol kinase A (*DGKA*), diacylglycerol kinase B (*DGKB*), phospholipase D1 (*PLD*1), phospholipase D3 (*PLD*3), membrane bound O-acyltransferase 1 (*MBOAT*1), and 1-acylglycerol-3- phosphate O-acyltransferase 2 (*AGPAT*2). Expression of the AGPAT2 gene was found to be upregulated in PRAD6, and the AGPAT4 gene showed upregulated levels in IMAD6, which could help promote PA accumulation. Correspondingly, expression levels of more types of PA were upregulated in PRAD6, and less PA upregulation was found in IMAD6, which might be related to the tendency of different types of ATGAP exhibiting the necessity of different fatty acyl-CoA donors [30]. When *PLD*1 and *PLD*3 expression levels are upregulated in PRAD6, this process promotes the conversion of phosphatidylcholine (lecithin) to PA, which also partly leads to the production of more types of PA in PRAD6 than lecithin. CDP-diacylglycerol synthase 1 (*CDS*1) expression is upregulated in IMAD6, which promotes the conversion of PA to CDP-diacylglycerol, and further reduces the proportion of PA subjected to upregulation in IMAD6. Choline phosphotransferase 1 (*CHPT*1) catalyzes the synthesis of phosphatidylcholine from CDP-choline [31]. It was also observed that lecithin expression was more upregulated in IMAD6 than in PRAD6. Lecithin-cholesterol acyltransferase (*LCAT*), lysophosphatidylcholine acyltransferase (*LPCAT*), phospholipase A and acyltransferase 3 (*PLA*2*G*16), and phospholipase B1 (*PLB*1) exhibit reactions with lecithin (phosphocholine) and 1-acyl-sn-glycero-3-phosphocholine (LPA) substrates. High expression in PRAD6 is likely to reduce the concentration of its reaction substrate, resulting in the upregulated expression of lecithin and phosphocholine in IMAD6 compared to that occurring in PRAD6.

Expression levels of *DGKA, DGKB, PLD*1, *PLD*3, *MBOAT*1, and *AGPAT*2 genes were found to be upregulated in PRAD6, which promoted the accumulation of PA in PRAD6. Among them, *DGK*, acting as a lipid kinase, can metabolize diacylglycerol (DG) into PA [32]. The PLD gene encodes an intracellular enzyme that produces PA by catalysing the hydrolysis of PC in response to extracellular signals [33]. MBOAT1 is a lyso-phosphatidylserine (lyso-PS) acyltransferase that can catalyse the acylation of a variety of substrates and the transfer of acyl groups from acyl-CoA to lysophospholipid to produce PA. It also participates in the reacylation step (Lands cycle) of the phospholipid remodelling pathway [34]. AGPAT1 catalyzes the production of PA with lysophosphatidic acid (LPA) and acyl-CoA as substrates [30]. Consistent with this, *CDS*1 level was found to be upregulated in IMAD6, which reduced the proportion of PA upregulated in IMAD6. CDS1 catalyzes the conversion of PA into CDP-diacylglycerol (CDP-DAG), which is an essential intermediate for the synthesis of phosphatidylglycerol, cardiolipin, and phosphatidylinositol [35], and actively regulates the differentiation and development of adipocytes [36]. The high expression of *LCAT, LPCAT, PLA*2*G*16, and *PLB*1 in PRAD6 concentrates their reaction substrates, resulting in PC showing the existence of more upregulated species in IMAD6 than those observed in PRAD6.

Transcriptomics and proteomics studies have shown that the combined effects of increased fat formation, fatty acid intake, and fatty acid esterification, as well as decreased lipolysis, are related to increased IMF deposition in the longissimus dorsi (LM) [37]. In differentiated adipocytes, the total SFA content in pig subcutaneous adipocytes is significantly higher than that in intramuscular adipocytes, and the unsaturated fatty acid (including MUFAs and PUFAs) contents in intramuscular adipocytes tends to be higher than that in subcutaneous adipocytes [38]. During the comparison of the fatty acid content between longissimus dorsi fat and perirenal fat in three breeds, Holstein cattle, Simmental cattle, and Chinese Longdong cattle, a general difference in the percentage of fatty acids between the two fat storage parts was found. The percentages of PUFAs and MUFAs in longissimus dorsi fat were found to be higher, and the proportion of n6:n3 PUFA was higher. Furthermore, the longissimus dorsi Δ9-desaturase catalytic activity index was higher than that of prerenal fat [39]. Similar research has been conducted on processed raw materials. In beef, perirenal fat had a higher total SFA content than intermuscular fat, but the total USFA content in intermuscular fat was higher [40].

Thus far, studies have been conducted to compare adipocytes derived from perirenal, subcutaneous, and intramuscular regions. Although a few significant differences in gene expression have been observed between subcutaneous and perirenal adipocytes, the differences between IMF and non-muscle adipocytes are even more significant [2]. In agreement with previous findings reported in pigs [2], we found that LPL, PPARG, and SREBF1 (the genes involved in the transcriptional regulation of adipogenesis, TG hydrolysis, and lipid metabolism) exhibited lower gene expression levels in the IMF than those observed in the perirenal adipocytes. Meanwhile, the gene expression level of IGF2 was higher in the IMF. In contrast to Gardan’s [2] study conducted on pigs, studies on cattle showed that FASN was expressed at a higher level in the IMA [37,41]. Our research also supports this finding. Studies have shown that the expression of FASN, related to fatty acid synthesis, is positively correlated with the IMF content of beef cattle [37]. Notably, similar to results of a study reported by Sheng et al [42], our study found that the CIQTNF family was highly expressed in IMF, especially CIQTNF3, which is a gene product of adipocyte differentiation. Moreover, it demonstrated structural similarities with adiponectin in adipocytes [43] and upregulated the secretion of adiponectin [44], which might be involved in metabolism and immune pathways.

## 5. Conclusions

This study highlights a combined transcriptomic and metabolomic analysis conducted for elucidating the mechanisms of perirenal and intramuscular fat deposition in beef cattle. First, Qinchuan cattle perirenal preadipocytes and intramuscular preadipocytes were isolated and identified. PRA were found to possess a higher adipogenic ability, whereas IMA possessed a higher proliferation ability. Based on the analysis of differentially expressed genes, we determined that different signalling pathways regulated proliferation and differentiation in the perirenal preadipocytes and intramuscular preadipocytes. Finally, using the data extracted based on the conduction of lipidome and transcriptome analysis, IMA were found to contain more unsaturated fatty acids than PRA, and this was associated with different lipid metabolism-related pathways and corresponding metabolites. Our findings will provide a research basis for screening the key metabolic pathways or genes and metabolites involved in intramuscular fat production in livestock. Additionally, the present study provides a theoretical basis for the discovery of adipocyte differentiation markers and for targeting the pathways involved in the regulation of beef cattle fat deposits in different parts of the body.

## Supplementary Material

Supplemental Material

## Data Availability

The data presented in this study are available in the Sequence Read Archive (SRA) database, accession number: PRJNA775885- (https://www.ncbi.nlm.nih.gov/sra/PRJNA775885).
