# Supplementary material for: RNA-Seq and lipidomics reveal different adipogenic processes between bovine perirenal and intramuscular adipocytes
Source: Adipocyte. 2022 Aug 8;11(1):448–62. doi: 10.1080/21623945.2022.2106051 (PMC9367662; doi:10.1080/21623945.2022.2106051)
Supplement: Supplemental Material [file KADI_A_2106051_SM8327.zip › supplementary/Figure Legends.docx]

**Figure 1.** A scheme of chronological preadipocyte differentiations and sampling time points. After the induction of adipogenic differentiation by DMI, the contact-suppressed preadipocytes underwent several stages of differentiation, namely early, intermediate, and late stages. The day of induction was defined as Day 0. (5-ethynyl-2’-deoxyuridine) EdU assays were performed one day before induction(Day-1). EdU assays, cell immunofluorescence, and RNA-seq were performed 16 h after induction, and the groups were named PRAMCE and IMAMCE. Oil red o staining, cell immunofluorescence, RNA-seq, and lipidomic assays were performed on the 6th day after induction, and the groups were named PRAD6 and IMAD6.

**Figure 2.** Preadipocyte identification (magnification: 100×). DAPI (blue) staining was performed to investigate the nucleus, and the right panel shows the merged view. A. ZNF423 (red) immunofluorescence in perirenal preadipocytes (PRAs). B. ZNF423 (red) immunofluorescence in intramuscular preadipocytes (IMAs). C. PPARG (red) immunofluorescence in PRAs. D. PPARG (red) immunofluorescence in IMAs. E. Presence of perirenal adipocyte lipid droplets and the results of oil red O staining. F. Presence of intramuscular adipocyte lipid droplets and the results of oil red O staining. G. Statistical results for an oil red O-stained area. H. Adiponectin (ADPN) immunofluorescence of PRAD6. I. ADPN immunofluorescence of IMAD6. DAPI (blue) staining was performed to investigate the nucleus, and the right panel shows the merged view. J. Percentage statistical results for ADPN-immunoreactive cells. Double asterisks (**) indicate highly significant variations (*p* < 0.01). Error bars represent SD (n = 3).

**Figure 3.** Analysis of the differentially expressed genes (DEGs) between perirenal preadipocytes (PRAMCE) and intramuscular preadipocytes (IMAMCE) during MCE. A, B. EdU assays conducted using perirenal preadipocytes/intramuscular preadipocytes (PRAs/IMAs) before confluency was achieved (Day-1, magnification: 100×). Cells during DNA replication were stained with EdU (red), and the nuclei were stained with the Hoechst stain (blue). C, D. EdU assays conducted using PRAs/IMAs after differentiation (MCE, magnification: 100×). E. Percentage statistical results for positive EdU cells before confluency was achieved (Day-1). F. Percentage statistical results for positive EdU cells after differentiation (MCE). Double asterisks (**) indicate highly significant variations (*p* < 0.01). Error bars represent SD (n = 3). G. DEGs observed between PRAMCE and IMAMCE during MCE. H. qPCR validation of the selected DEGs. I. KEGG enrichment of DEGs in the PRAs and IMAs during MCE.

**Figure 4.** Adipocyte proliferation and adipogenesis in perirenal preadipocytes (PRAs) and intramuscular preadipocytes (IMAs) are regulated by different pathways during the specific cell division phase (MCE). A. Adipocyte proliferation in PRAMCE and IMAMCE is regulated by different pathways during MCE. B. Adipocyte adipogenesis in PRAMCE and IMAMCE is regulated through different pathways.

**Figure 5.** Adipocyte differentiation of PRAs and IMAs are regulated by different pathways. A. Differentially expressed genes (DEGs) during PRA differentiation. C. DEGs during IMA differentiation. E. DEGs between PRAD6 and IMAD6. B, D. F. qPCR validation of the selected DEGs. G. The KEGG enrichment pathway of the two groups of differential genes during PRA differentiation. H. The KEGG enrichment pathway of the two groups of differential genes during IMA differentiation.

**Figure 6.** Functional analysis of the differentially expressed genes in bovine perirenal adipocytes (PRAD6) and intramuscular adipocytes (IMAD6) on the 6th day after differentiation. A. GO function enrichment analysis of DEGs upregulated in PRAD6. B. GO function enrichment analysis of DEGs upregulated in IMAD6. C. Enrichment of DEGs between PRAD6 and IMAD6 in pathways related to adipogenic differentiation and metabolism. The yellow circle indicates the enriched pathway. The red circles indicate genes upregulated in PRAD6, and the green circles indicate genes upregulated in IMAD6. The darker the color, the greater the absolute value of log2FC. The larger the circle of a single gene, the more pathways that related to it.

**Figure 7.** Lipid composition and changes detected in PRAD6 and IMAD6. A. Lipid contributions. B. Volcano map for the differential metabolites. C. Descriptive statistics for the different metabolites. D. Changes in metabolites between the two groups. E. Annotation to the KEGG database for significantly different metabolites (*p* ≤ 0.05). F. Composition analysis of the acyl chains of the FFAs. The abscissa indicates the number of carbon atoms in the acyl chain. The ordinate indicates the fold change of perirenal preadipocytes (PRAs) relative to intramuscular preadipocytes (IMAs). A log2fold-change > 0 indicates a metabolite with upregulated expression in the PRAD6 group, whereas a log2fold-change < 0 indicates that the metabolite expression was upregulated in the IMAD6 group. G. Double bond analysis of FFAs. The abscissa indicates the number of unsaturated bonds in the acyl chain. The ordinate indicates the fold change of PRAD6 relative to IMAD6.

**Figure 8.** Comparison of glycerolipid, glycerophospholipid, and sphingomyelin compositions between bovine perirenal adipocytes and intramuscular adipocytes. A-B. Differential genes and metabolites in the pathway. C-E Composition analysis of the acyl chains of PE, PC, PS, PI, PG, CER, and SM. The abscissa represents the number of carbon atoms in a single fatty acyl chain related to different glycerophospholipids or sphingolipids. The ordinate represents the fold change of perirenal preadipocytes (PRAD6) relative to the intramuscular preadipocytes (IMAD6).
