# Supplementary figures and images for: RNA-Seq and lipidomics reveal different adipogenic processes between bovine perirenal and intramuscular adipocytes
Source: Adipocyte. 2022 Aug 8;11(1):448–62. doi: 10.1080/21623945.2022.2106051 (PMC9367662; doi:10.1080/21623945.2022.2106051)

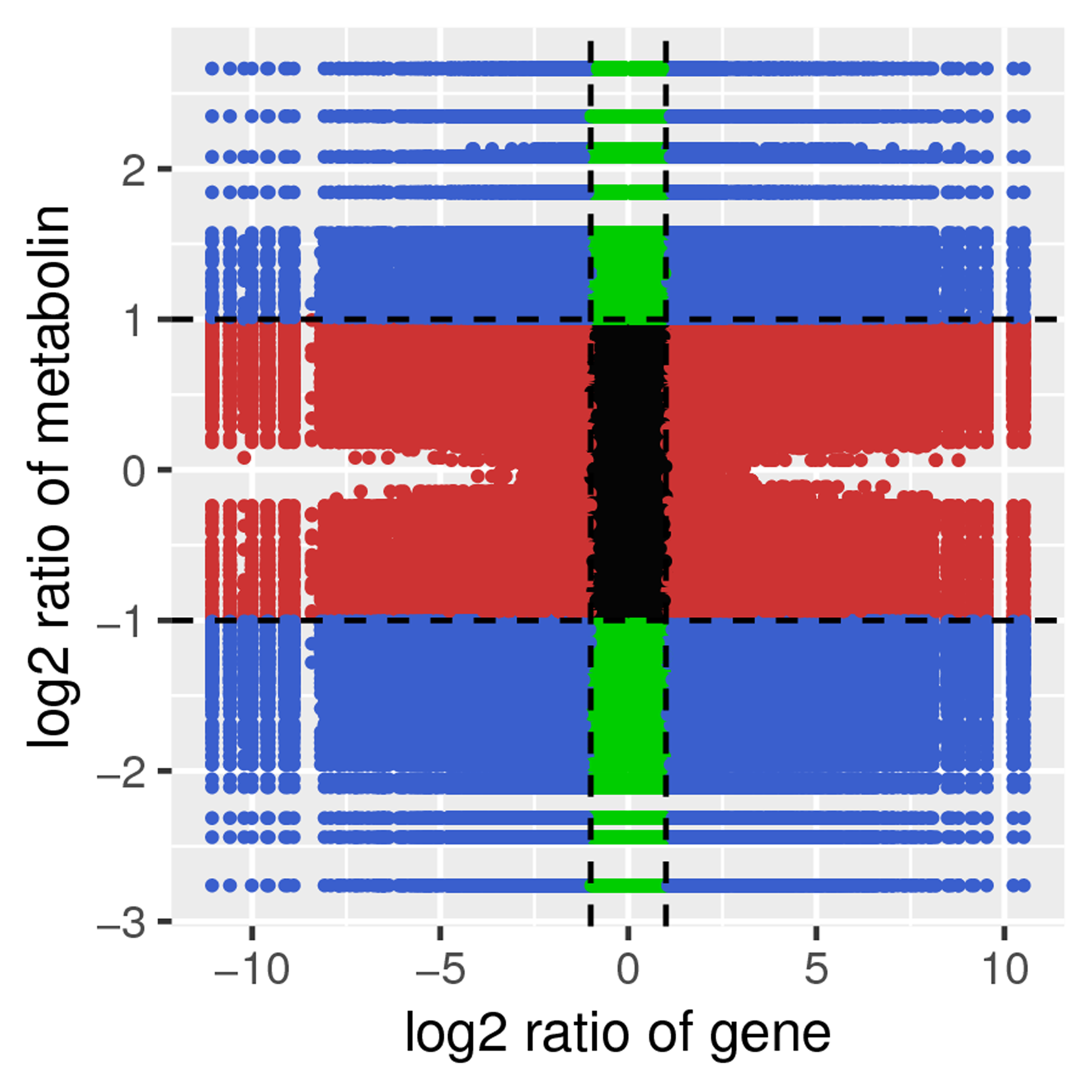

Supplement: Supplemental Material [file KADI_A_2106051_SM8327.zip › supplementary/Figure S1.tif]

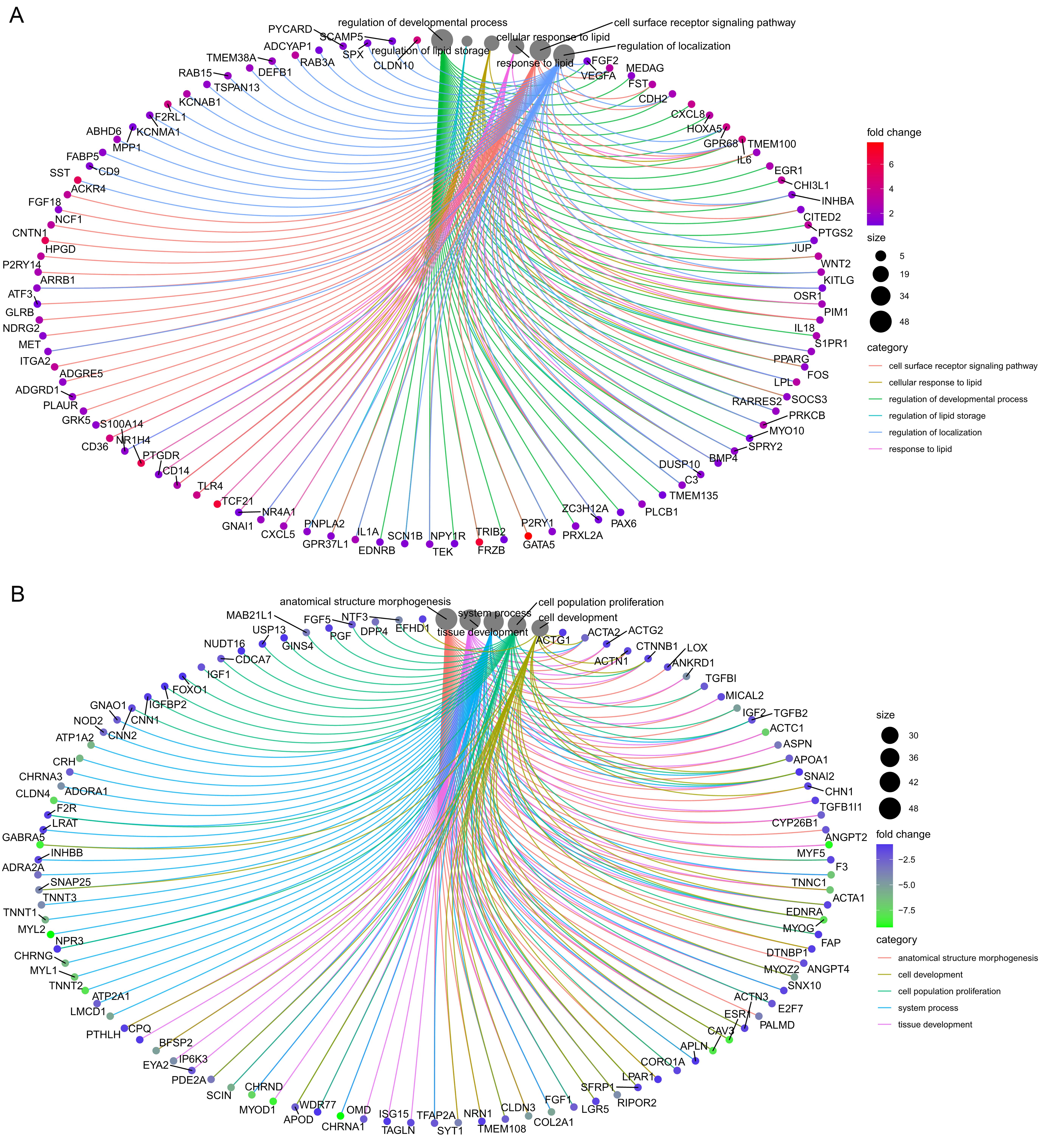

Supplement: Supplemental Material [file KADI_A_2106051_SM8327.zip › supplementary/Figure S2.tif]

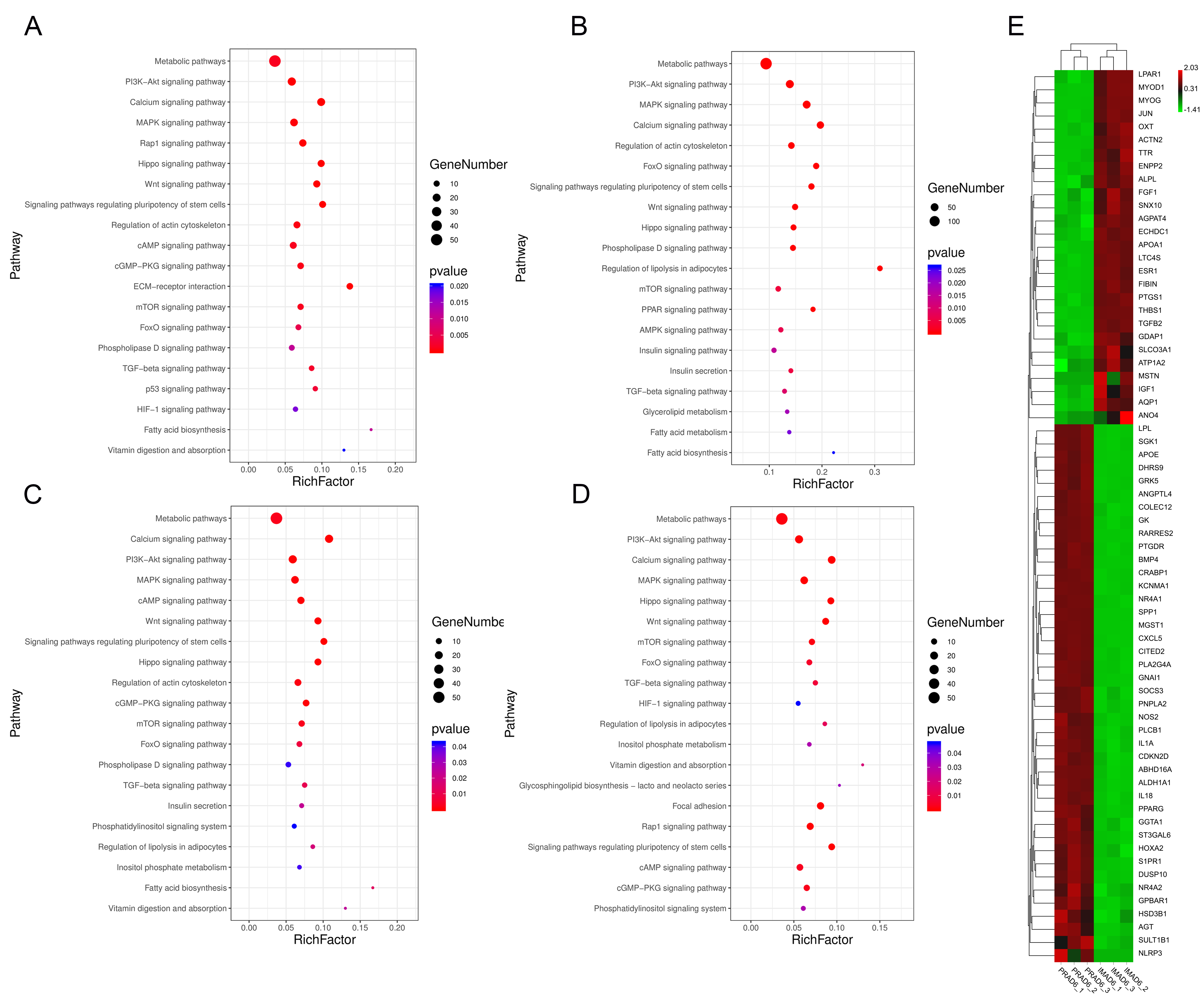

Supplement: Supplemental Material [file KADI_A_2106051_SM8327.zip › supplementary/Figure S3.tif]
